# Blood Donors' Preferences Toward Incentives for Donation in China

**DOI:** 10.1001/jamanetworkopen.2023.18320

**Published:** 2023-06-14

**Authors:** Yu Wang, Peicong Zhai, Shan Jiang, Chaofan Li, Shunping Li

**Affiliations:** 1 Center for Health Management and Policy Research, School of Public Health, Cheeloo College of Medicine, Shandong University, Jinan, Shandong, China; 2 NHC Key Lab of Health Economics and Policy Research, Shandong University, Jinan, Shandong, China; 3 Center for Health Preference Research, Shandong University, Jinan, Shandong, China; 4 Blood Center of Shandong Province, Jinan, Shandong, China; 5 Macquarie University Centre for the Health Economy, Macquarie Business School and Australian Institute of Health Innovation, Macquarie University, Sydney, New South Wales, Australia

## Abstract

**Importance:**

Blood donation is critical for health care systems, but donor retention remains challenging. Understanding donors’ preferences can inform incentive design and improve retention rates.

**Objective:**

To identify donor preferences for incentive attributes and their relative importance in promoting blood donation among Chinese donors in Shandong.

**Design, Setting, and Participants:**

This survey study fielded a discrete choice experiment (DCE) with a dual response design among blood donors, analyzing the responses under forced and unforced choice settings. The study took place from January 1 to April 30, 2022, in 3 cities (Yantai, Jinan, and Heze) representing diverse socioeconomic strata in Shandong, China. Eligible participants were blood donors aged 18 to 60 years who had donated within the preceding 12 months. Participants were recruited using convenience sampling. Data were analyzed from May to June 2022.

**Exposure:**

Respondents were presented with different blood donation incentive profiles, varying in health examination, blood recipient, honor recognition, travel time, and gift value.

**Main Outcome and Measure:**

Respondent preferences for nonmonetary incentive attributes, attribute relative importance, willingness-to-discard values for attribute improvement, and estimated uptake of new incentive profiles.

**Results:**

A total of 650 donors were invited, of which 477 were included for analysis. The respondents were predominately male (308 respondents [64.6%]), aged 18 to 34 years (291 respondents [61.0%]), and had undergraduate degrees or higher (286 respondents [59.9%]). Among the 477 valid respondents, the mean (SD) age was 31.9 (11.2) years. Respondents preferred comprehensive health examination, family members as recipients, central government recognition, 30-minute travel time, and a gift valued at 60 Renminbi (RMB). No significant differences were found between the model results of forced and unforced choice setting. Blood recipient was the most important attribute, followed by health examination and gifts, and then honor and travel time. Respondents were willing to discard RMB 32 (95% CI, 18-46) for an improved health examination and RMB 69 (95% CI, 47-92) for changing the recipient from themselves to family members. Scenario analysis estimated 80.3% (SE, 0.024) of donors would endorse the new incentive profile if the recipient was changed from themselves to family members.

**Conclusions and Relevance:**

In this survey study, blood recipient, health examination, and gift value were perceived more important as nonmonetary incentives than travel time and honor recognition. Tailoring incentives according to these preferences may improve donor retention. Further research could help refine and optimize incentive schemes for blood donation promotion.

## Key Points

**Question:**

What are the preferences of blood donors toward nonmonetary incentives, and how can these preferences inform the design of effective incentives to encourage blood donation?

**Findings:**

This survey study of 477 blood donors identified the blood recipient as the most significant factor in motivating donations. Respondents prioritized designating family members as recipients and valued comprehensive health examinations and higher gift values; distinct preference patterns emerged among various donor subgroups.

**Meaning:**

Understanding donor preferences can inform the design of targeted, nonmonetary incentive policies that effectively encourage blood donations and contribute to self-sufficiency in safe blood and blood products.

## Teaser

This survey study evaluates nonmonetary incentives for blood donation in Shandong Province, China.

## Introduction

Blood transfusion constitutes an integral facet of health care, serving a crucial function in preserving lives and enhancing the quality of life for countless individuals worldwide.^1^ Although some countries have well-structured health systems that consistently meet the demand for blood products, many developing countries struggle with the persistent issue of chronic blood shortages.^2^ In 2021, China's whole blood donation rate was 12 donations per 1000 individuals, barely satisfying the nation's essential blood demand.^3,4^ The demand continues to grow in tandem with the rapid growth of health care service requirements among China's aging populace.^5^ Concurrently, increasingly stringent donor selection criteria have led to a contraction in the pool of eligible donors.^2^ The shrinking blood donor pool is unable to adequately address the escalating demand for clinical blood use, rendering the procurement of a sufficient, safe, and sustainable blood supply a significant challenge for China.^6,7,8^

Donation incentives, frequently described as extrinsically motivated encouragement intended to stimulate donation behavior, can manifest in either monetary or nonmonetary forms.^9^ Empirical evidence suggests that monetary incentives possess the potential to crowd out donors' intrinsic motivations, thereby attenuating blood donation behaviors.^10^ By contrast, some studies have described the positive association of nonmonetary incentives with blood donation.^11,12,13^ In 2018, 64 countries collected most (99% or more) of their blood supply through voluntary nonremunerated blood donation mechanisms.^4^ As a country with a vast population of 1.4 billion, China confronts unique challenges in establishing a voluntary nonremunerated donation system, primarily due to traditional beliefs that foster reluctance toward blood donation.^14^

Although the nonmonetary incentives may exert a positive impact on blood donation behavior, empirical evidence remains limited. Research is needed for comprehending the utility of nonmonetary incentives for blood donation within the context of a voluntary nonremunerated system. The discrete choice experiment (DCE) is an established stated preference approach, designed to simulate the impact of various attributes of a good or service on individual choice.^15,16^ In a DCE, respondents are asked to choose between 2 or more hypothetical incentive scenarios specified by several dimensions (called *attributes*) that differ in their settings (called *levels*) between the alternatives.^15^ This method is particularly useful for quantifying preferences for nonmarket goods and services such as voluntary blood donation. Only 1 study used DCE among college students to investigate preferences for blood donation incentives,^17^ the results of which could not be extrapolated to the Chinese population due to different contexts and an irrelevant respondent sample. Therefore, we conducted a DCE to elicit preferences for nonmonetary incentives among blood donors in Shandong province, China, aiming to generate policy implications for the establishment of a voluntary nonremunerated donation system in the region.

## Methods

The DCE was conducted in Shandong between January and April 2022, with approval from the ethics committee of the Center for Health Management and Policy Research, Shandong University. Before participating in the survey, all respondents provided written informed consent. Our study adhered to the checklist developed by the International Society for Pharmacoeconomics and Outcomes Research (ISPOR) reporting guideline for good practices pertaining to DCE in health care.^18^

### Identification of Attributes and Levels

The DCE commenced with the identification of attributes of the nonmonetary incentives for blood donation and defining their respective levels. We primarily extracted the factors from prior literature (eTable 1 in Supplement 1).^9,10,11,12,13^ This was followed by in-depth interviews with 24 blood donors (eTable 2 and eTable 3 in Supplement 1) and a focus group meeting with 4 survey practitioners and 4 blood donation professionals in Jinan, Shandong (eTable 4 in Supplement 1), to validate the attributes and finalize their levels. Ultimately, the DCE questionnaire included 5 attributes: (1) health examination types, (2) designated blood donation recipients, (3) donation honor, (4) travel time to donation stations, and (5) monetary value of donation gifts. Table 1 provides detailed attribute and level descriptions.

**Table 1.** Attributes and Associated Levels Identified for This Discrete Choice Experiment

Attribute (abbreviation)	Level	Level abbreviation
Health examination (examination)	Blood test only [Reference]	Blood
	Standard examination on selected items	Standard
	Comprehensive examination	Comprehensive
Blood recipient (recipient)	Future blood recipients unknown [Reference]	Unknown
	Future blood recipients are the donors themselves	Self
	Future blood recipients are family members	Family
Honor for donation (honor)	Certificate from the blood donation stations [Reference]	Certificate
	Honors or recognition from workplace (or school for student donors)	Workplace
	Honors or recognition from the central government	Government
Travel time (travel)	90-min travel time from home/workplace to the nearest blood donation station [Reference]	90 min
	60-min travel time from home/workplace to the nearest blood donation station	60 min
	30-min travel time from home/workplace to the nearest blood donation station	30 min
Gift (gift)	Gift with a monetary value equivalent to RMB 20 [Reference]	RMB 20
	Gift with a monetary value equivalent to RMB 40	RMB 40
	Gift with a monetary value equivalent to RMB 60	RMB 60

### Experimental Design and Questionnaire Development

**Figure 1.**
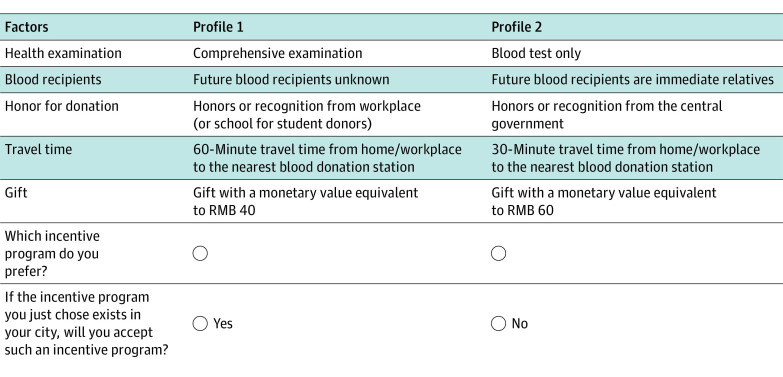
A Choice Task Example in the Discrete Choice Experiment

A fractional factorial design was executed using SAS version 9.4 (SAS Institute), resulting in 18 choice tasks. To mitigate the cognitive burden for respondents,^19^ the 18 tasks were divided into 3 blocks of 6 tasks each, with respondents randomized into 1 of the 3 blocks. We used a dual response choice design.^20^ Each choice task contained a forced and unforced choice task (Figure 1; eFigure 1 in Supplement 1). In the forced task, respondents made a choice between 2 hypothetical profiles. The unforced task provided an opt-out option, requiring respondents to choose between the chosen profile from the forced task and the opt-out option.

The questionnaire comprised an introduction to the research background and attribute definitions, 6 DCE choice tasks, and sociodemographic inquiries. Before the formal choice tasks, a practice task familiarized respondents with subsequent choice tasks. A duplicated choice task was inserted to ensure respondent attentiveness and consistent choices (ie, consistency test). Collected sociodemographic information encompassed sex, age, education, occupation, donation experience, donation volume, and volunteer type. Following the questionnaire finalization, we conducted pilot testing among donors to verify the comprehensibility and acceptability of the questionnaire, making modifications according to feedback to enhance clarity. Further details are available in the eAppendix in Supplement 1.

### Data Collection

Data were collected from January 1 to April 30, 2022. Eligible participants were blood donors aged between 18 and 60 years who had donated within the preceding 12 months. To ensure sample representativeness for the donor population in Shandong, participants were recruited from 3 cities—Yantai, Jinan, and Heze—located in the eastern, central, and western regions of the province, representing diverse socioeconomic strata. In each city, a blood donation station and a mobile donation vehicle were selected. A convenience sampling approach was used to recruit donors who visited the selected donation facilities when we surveyed. The sampling process combined predetermined sex and age quotas according to the 2018 China Report on Blood Safety to enhance sample representativeness.^5^

According to the pilot test process and results, the minimum completion time for the questionnaire was determined to be 120 seconds. If a respondent chose different responses in the 2 duplicated choice tasks (consistency test), and submitted the questionnaire within 120 seconds, they were considered for removal in the final analysis.

Trained interviewers conducted face-to-face data collection at recruitment locations. The minimum sample size required for ensuring statistical power was determined by the rule of thumb.^21^ The interviewers alternated between the 3 versions of the questionnaire during interviews to balance the sample size distributed between the 3 versions.

### Statistical Analysis

Two mixed logit (MIXL) models were used to analyze the forced choice data and the unforced choice data, respectively, in STATA version 15.1 (StataCorp).^22,23^ An alternative-specific constant was included in the MIXL for unforced data, indicating the utility generated by the opt-out option relative to the non–opt-out options. We used effects coding for categorical attributes.^24^ The mean of a parameter signified the average preference value (called *part-worth utility*) that donors attributed to a specific attribute level, and the standard deviation characterized the heterogeneity of the preference value among donors. To ensure the reliability of the parameter estimates, we iteratively estimated the MIXL models by incrementally increasing the number of random draws.

We applied nonparametric^25^ and parametric^26^ approaches to test for dominant preference. The attribute relative importance was calculated using the model estimates of the unforced choice data set through a commonly used rescaling method.^27^ All potential interactions between the characteristics of respondents and attribute levels were scrutinized through the use of multinomial logit models.^15^ After the identification of interaction terms, we simulated a MIXL model including the interactions. We conducted subgroup analyses by age, sex, education level, first-time donor, and individual volunteer.

We calculated the marginal rate of substitution between gift value and other aspects of nonmonetary incentives and interpreted the results as donors’ willingness-to-discard (WTD) gifts for the improvement of other incentive factors. By comparing the WTD values for the improvement of 4 factors, policy implications were generated for efficiently augmenting nonmonetary incentives to elevate the utility of donors.

The prevailing incentive profile in Shandong comprised providing blood tests and certificates to donors, designating donors themselves as the recipients of their donation, and a mean 90-minute travel time to the nearest donation station. We constructed hypothetical incentive profiles where the attributes were improved compared with the base profile. The comparisons between the hypothetical profiles and the base profile constituted the scenario analysis. The outcome of scenario analysis was denoted by the estimated uptake for each hypothetical profile relative to the base case, defined as the percentage of donors amenable to supporting the new incentive profile, which showcased how the improvement endeavor could motivate donors. The 95% CIs for estimated uptake were generated using the delta method.^28^ Data were analyzed from May to June 2022.

## Results

### Pilot Testing

A total of 86 donors completed the questionnaire for pilot testing. Model estimates exhibited signs and order as we expected (eTable 5 in Supplement 1). These results indicated that respondents understood the DCE choice tasks and could manage the number of questions.

### Respondent Characteristics

A total of 650 donors were invited to participate in the survey, of which 570 participated (response rate 87.7%) and finally, 568 completed the questionnaire. The mean (SD) completion time was 5.5 (1.68) minutes. A total of 91 respondents who failed the consistency check and completed the questionnaire within 120 seconds were removed from the analysis. The remaining 477 respondents were included in the analysis, comprising 106 respondents from Yantai, 252 from Jinan, and 119 from Heze. Among the 477 valid respondents, the mean (SD) age was 31.9 (11.2) years. Table 2 presents the 477 participants’ sociodemographic characteristics in comparison with the donor population statistics in Shandong according to the 2018 official report.

**Table 2.** Demographics of Respondents Included for Analysis

Characteristic	No. (%)				2018 China Report on Blood Safety, %
	All respondents (N = 477)	Yantai city (n = 106)	Jinan city (n = 252)	Heze city (n = 119)	
Sex					
Male	308 (64.6)	60 (56.6)	186 (73.8)	62 (52.1)	64
Female	169 (35.4)	46 (43.4)	66 (26.2)	57 (47.9)	36
Age, y					
18-24	173 (36.3)	34 (32.1)	105 (41.7)	34 (28.6)	29
25-34	118 (24.7)	27 (25.5)	64 (25.4)	27 (22.7)	27
35-44	97 (20.3)	21 (19.8)	43 (17.1)	33 (27.7)	25
45-60	89 (18.7)	24 (22.6)	40 (15.9)	25 (21.0)	19
Education					
Primary school or below	11 (2.3)	7 (6.6)	2 (0.8)	2 (1.7)	NA
Secondary school	80 (16.8)	22 (20.8)	34 (13.5)	24 (20.2)	NA
High school	100 (21.0)	30 (28.3)	32 (12.7)	38 (31.9)	NA
Undergraduate	263 (55.1)	45 (42.5)	166 (65.9)	52 (43.7)	NA
Graduate or above	23 (4.8)	2 (1.9)	18 (7.1)	3 (2.5)	NA
Occupation					
Agriculture and industry	130 (27.3)	36 (34.0)	63 (25.0)	31 (26.1)	NA
Student	113 (23.7)	16 (15.1)	74 (29.4)	23 (19.3)	NA
Government/public service	34 (7.1)	20 (18.9)	1 (0.4)	13 (10.9)	NA
Health care professional	26 (5.5)	4 (3.8)	21 (8.3)	1 (0.8)	NA
Private company	77 (16.1)	18 (17.0)	43 (17.1)	16 (13.4)	NA
Others^a^	97 (20.3)	12 (11.3)	50 (19.8)	35 (29.4)	NA
Donation experience					
First-time donor	182 (38.2)	23 (21.7)	113 (44.8)	46 (38.7)	NA
Repeated donor^b^	295 (61.8)	83 (78.3)	139 (55.2)	73 (61.3)	NA
Blood volume donated					
200 mL	84 (17.6)	2 (1.9)	75 (29.8)	7 (5.9)	NA
300 mL	29 (6.1)	12 (11.3)	0	17 (14.3)	NA
400 mL	364 (76.3)	92 (86.8)	177 (70.2)	95 (79.8)	NA
Volunteer type^c^					
Individual volunteer	315 (66.0)	101 (95.3)	101 (40.1)	113 (95.0)	NA
Group volunteer	162 (34.0)	5 (4.7)	151 (59.9)	6 (5.0)	NA

Abbreviation: NA, not available.

^a^ Others: self-employed, freelancers, unemployed, retirees, or unknown.

^b^ Repeated donor: 2 or more donations before the survey.

^c^ Individual volunteer: self-motivated and donated solely based on personal choice.

The respondents were predominately male (308 respondents [64.6%]), young adults aged between 18 and 34 years (291 respondents [61.0%]), and with undergraduate degrees or above (286 participants [59.9%]). The vast majority donated 400 mL of blood (364 participants [76.3%]). Two-thirds of the donors (315 participants [66.0%]) were self-motivated (ie, individual volunteers), rather than motivated by their employers (ie, group volunteers).

We undertook a χ^2^ test between our total sample and the Shandong donor population and found no evidence of significant differences in terms of age and sex. The samples from the 3 cities had similar proportions in terms of age and sex. χ^2^ tests for age and sex between the 3 city samples did not reveal evidence of significant differences.

### Model Estimates

We found no evidence of dominant preference (eTable 6 in Supplement 1). Estimation stability was attained when 2500 random draws were used for the MIXL models of the forced and unforced choice data sets (eFigure 2, eFigure 3, eFigure 4, and eFigure 5 in Supplement 1), resulting in our final estimates.

The model estimates for forced choices (eTable 7 in Supplement 1) and unforced choices (eTable 8 in Supplement 1) indicate that respondents placed positive values on the best levels and negative values on the worst levels of the attributes, as we expected. Respondents were more likely to be stimulated for future donations by the provision of a comprehensive health examination, the designation of family members as the recipient of blood donation, honorable recognition by the central government, a travel time of 30 minutes to the nearest donation station, and a gift valued at RMB 60. They were less likely to be motivated by the provision of a blood test, the designation of unknown people as the recipient, the honorable recognition by the workplace, a travel time of 90 minutes, and a gift valued at RMB 20.

Notably, the coefficient for opt-out was −9.82 (95% CI, −12.60 to −7.04), suggesting that opting out incurred a negative part-worth utility for respondents. This result indicated that respondents placed a positive value on non–opt-out options and were less likely to discontinue donation in the future.

### Preference Heterogeneity

The standard deviations in the 2 MIXL models (forced and unforced, respectively) for most attribute levels were statistically significant, indicating preference heterogeneity. Consequently, some middle levels generated positive part-worth utility for some donors but negative part-worth utility for others. For example, in the model for unforced choices, 55.0% of respondents placed a negative value on the 60-minute travel time, while the remaining 45.0% placed a positive value on this attribute level.

### Forced Choices vs Unforced Choices

**Figure 2.**
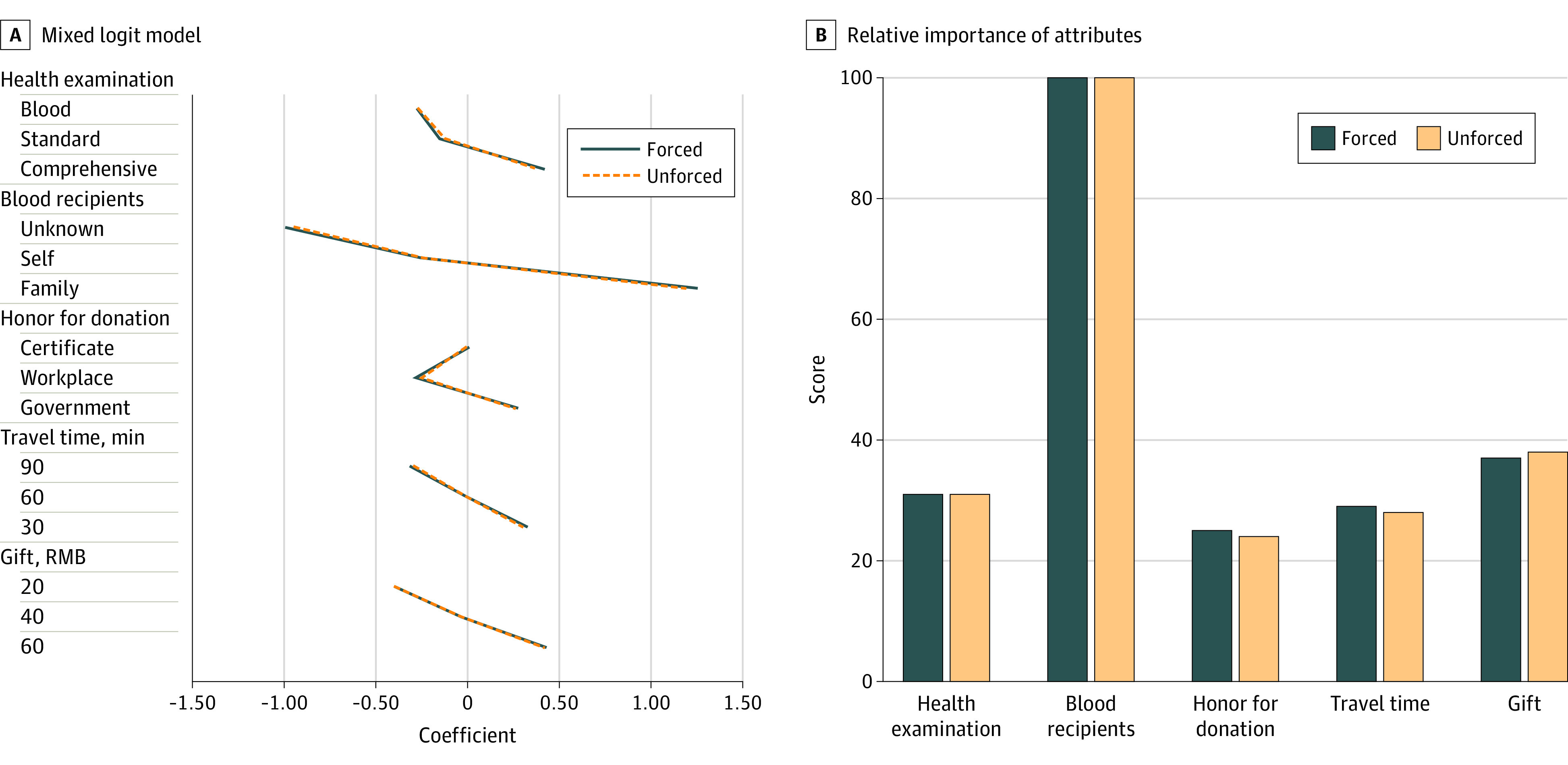
Coefficients of Mixed Logit Models and Relative Importance of Attributes Under Forced and Unforced Choice Settings The relative importance of each attribute was determined by dividing the range of coefficients within the attribute by the sum of all attribute ranges, subsequently rescaled on a 1 to 100 range. The highest value denoted the most important attribute perceived by the respondents.

We compared the model results between the forced choices and the unforced choices and found no significant differences (Figure 2). The preference values within each attribute between the 2 models were closely aligned, indicating that the inclusion of opt-out did not affect the choices significantly. The preference weights increased monotonically within 4 attributes, except that the middle level of honor (ie, “recognition by workplace”) had the lowest value compared with the other 2 levels.

### Attribute Relative Importance

We used both the forced and unforced choice models to derive the relative importance of attributes and found similar results (Figure 2; eTable 9 in the Supplement). After rescaling, the blood recipient was the most important attribute. Comparatively, the remaining 4 attributes were merely one-third as important as the blood recipient attribute. Among the 4 attributes, gifts and health examination were more important than honor and travel time. Therefore, we divided the attributes into 3 tiers with descending importance: tier 1 included recipient, tier 2 included health examination and gifts, and tier 3 consisted of honor and travel time.

### Willingness-to-Discard

According to the model estimates of unforced choices with the gift attribute coded as a continuous variable (eTable 10 in the Supplement), we derived respondents’ WTD values (eTable 11 in the Supplement), which indicate the monetary value of gifts that respondents were willing to discard for the improvement in the other attributes. On average, respondents were willing to discard RMB 32.03 (95% CI, 18.22-45.84) if the health examination type was improved from blood test to comprehensive examination. The WTD number doubled (RMB 69.18; 95% CI, 46.58-91.78) if the donation recipient was changed from donor themselves to family members, which was also the highest WTD value for the improvement in a single attribute. The WTD value comparison indicated that respondents valued the improvement in donation recipient more than that in other attributes.

### Interactions

We identified 3 significant interaction terms (eTable 12 in the Supplement). The first interaction was between the male indicator and the level self in the recipient attribute. The negative value indicated that, compared with female participants, male participants were less likely to designate themselves as the recipient of the donation. The second interaction was between education level and the attribute level self. The positive value implied that respondents with a high school education or less were more likely to make themselves the donation recipient, compared with people with an undergraduate education or above. The third interaction was between the first-time donor and the 30-minute travel time. The positive value indicated that first-time donors, compared with repeat donors, were more likely to favor a short travel time.

### Scenario Analysis

Table 3 presents the results of the scenario analysis. In comparison with the base profile, 53.7% (SE, 0.026) of donors would support the new incentive profile if the health examination was improved from blood test to standard examination, all else being equal. The estimated uptake was the lowest among all comparisons. Conversely, 80.3% (SE, 0.024) of donors were estimated to endorse the new incentive profile if the recipient was changed from donor themselves to family members. A doubled gift value (ie, RMB 40) would attract 60.0% (SE, 0.016) of donors in comparison with the base case, and the uptake would increase to 69.2% (SE, 0.028) if the gift value was tripled.

**Table 3.** Estimated Uptake of Hypothetical Incentive Profiles Compared With Base Profile

Incentive	Base profile	Profile 1	Profile 2	Profile 3	Profile 4	Profile 5	Profile 6	Profile 7	Profile 8
Health examination	Blood	Standard	Comprehensive	Blood	Blood	Blood	Blood	Blood	Blood
Blood recipients	Self	Self	Self	Family	Self	Self	Self	Self	Self
Honor for donation	Certificate	Certificate	Certificate	Certificate	Government	Certificate	Certificate	Certificate	Certificate
Travel time, min	90	90	90	90	90	60	30	90	90
Gift, RMB	20	20	20	20	20	20	20	40	60
Estimated uptake of hypothetical profile, No. (SE)	NA	0.537 (0.026)	0.657 (0.026)	0.803 (0.024)	0.563 (0.027)	0.568 (0.025)	0.644 (0.028)	0.600 (0.016)	0.692 (0.028)

Abbreviation: NA, not applicable.

### Subgroup Analysis

Although all subgroups placed the greatest preference value on the recipient attribute, divergent preference patterns emerged between subgroups concerning the other 4 attributes (eFigure 6, eFigure 7, eFigure 8, eFigure 9, and eFigure 10 in Supplement 1). As demonstrated by the comparison between the young adults (age <35) and the middle-aged and older adults (aged 35 to 60), young adults perceived travel time as more important than health examination, whereas the middle-aged and older donors deemed health examination more important than travel time (eFigure 6 in Supplement 1). First-time donors considered travel time more significant than the other 3 attributes, while repeat donors placed the highest value on gifts (eFigure 9 in Supplement 1). A similar pattern arose when comparing individual and group volunteers (eFigure 10 in Supplement 1); while the former group prioritized gifts, the latter group regarded the gift as the least important attribute, as they were more concerned with travel time.

## Discussion

In this survey study, we investigated the preferences of blood donors in Shandong, China, providing insights into nonmonetary incentive factors that retain donors for future blood donation. Our findings revealed that the recipient of donated blood was the most crucial factor in motivating future donations, with gifts and health examinations also playing a significant role, surpassing the importance of donation honor and travel time. Notably, our estimations suggest that an improvement in the recipient category from the donor themselves to family members could garner support from more than 80% of donors. Furthermore, our analysis identified distinct preference patterns among various subgroups; for instance, young donors, first-time donors, and group volunteers placed a higher value on travel time than gifts, while middle-aged and older donors, repeat donors, and individual volunteers exhibited the opposite preference, deeming gifts more important than travel time. This study sheds light on the complex dynamics of blood donation preferences and underscores the importance of tailoring incentive policies.

Our study achieved a high response rate. Survey response rates may be affected by factors such as the complexity of the DCEs questionnaire, the survey mode, and respondents' familiarity with the topic.^29^ This study recruited samples from the donation facilities, where donors were familiar with donation incentives. Another factor was the face-to-face mode applied in this study. Although this approach may require higher monetary and time investments compared with online surveys, it can provide reliable high-quality data. Concurrently, the ISPOR guideline suggests that interviewer-led administration may improve the quality of data by explaining the task and answering questions.^30^ Face-to-face interviews are often recommended during the pretesting of attributes and levels.^31,32,33^ When designing this study, we deemed it necessary to use face-to-face interviews to help the respondents fully understand the questionnaire. We suggest that future studies use face-to-face interviewer-led administration to improve the data quality.

We used a dual response design in this study. The comparison of the results from the 2 models indicated that the addition of the opt-out option did not significantly change the respondents’ preferences. This finding contrasts with previous literature suggesting that restricted choice tasks may bias the analysis results.^16,34^ One possible explanation is that all respondents were donors who had already revealed their preference for blood donation. Therefore, when asked about their attitudes toward nonmonetary incentive improvement, at least some of them might have a strong aversion to the opt-out, leading them to adopt a semi-compensatory choice process with the non–opt-out alternatives constituting their actual consideration set.^35^ These participants made their choice among the alternatives within this consideration set following a utility maximization compensatory rule. If respondents of this type do exist, we may consider using the independent availability logit model to account for this type of choice behavior.^36,37^ In this study, we applied the MIXL model following the best practice recommendation by ISPOR and recognize that the independent availability logit model is still in its early stage of application in health care; we expect further exploration of its establishment and application in health care in future studies.

The finding that recipient was perceived as the primary consideration by donors is consistent with previous studies.^38,39,40^ The finding may be attributed to the fact that Chinese people place great importance on family and are willing to make sacrifices for their families.^41^ The finding suggests that expanding the scope of blood recipients to family members, such as the parents of donors, could effectively encourage future blood donations. Meanwhile, contrary to our expectation, we found that certificates from blood donation stations incurred higher part-worth utility than recognition by the workplace. We initially anticipated that respondents would value recognition by their workplace more than a certificate, which is consistent with a previous study that young donors did not value appreciation tokens in the form of a letter of gratitude or a card from the Blood Service.^10^ One possible explanation is that one-third of our respondents were group volunteers who were motivated by their superiors in their organizations or companies. They might have peer pressures to donate blood, which undermined the importance of recognition from their workplace and their intrinsic motivations.^14^

### Limitations

This study has some limitations. First, due to the sociodemographic heterogeneity between Shandong and other Chinese provinces, it is difficult to extrapolate our results to the entire Chinese donor population. Second, we are unaware of societal preferences due to our sampling approach, which is important for the establishment of a nonmonetary incentive system as the system aims at retaining donors and attracting individuals who have never donated blood. Future studies should investigate societal preferences concerning nonmonetary incentives.

## Conclusion

This study examined the preferences of blood donors in Shandong, China, with respect to nonmonetary incentives, providing insights for the development of effective blood donation incentive policies. Our findings indicate that the blood recipient is the most significant factor in motivating donations, followed by gifts and health examination type. Additionally, our analysis reveals diverse preference patterns among various donor subgroups, emphasizing the need for tailored incentive policies. A better understanding of donor preferences will facilitate the design of targeted, nonmonetary incentive policies that can effectively encourage blood donations and help achieve self-sufficiency in safe blood and blood products.

## Notes

Supplement 1. **eTable 1**. Literature Search Strategy **eTable 2. **Characteristics of Participants of Interviews **eTable 3. **Quotations Describing Blood Donors’ Perspectives on Candidate Attributes From Interviews **eTable 4. **Attributes and Levels Development From the Focus Group **eFigure 1. **Example Choice Task in the Original Chinese Version **eAppendix. **Supplementary Methods **eTable 5. **Multinomial Logit Model Results in the Pilot Study **eTable 6. **Multinomial Logit Models Result of Dominant Preference **eFigure 2. **Mixed Logit Model for the Forced Choice Dataset; Gift Attribute Was Coded as a Categorical Variable **eFigure 3. **Mixed Logit Model for the Unforced Choice Dataset; Gift Attribute Was Coded as a Categorical Variable **eFigure 4. **Mixed Logit Model for the Forced Choice Dataset; Gift Attribute Was Coded as a Continuous Variable **eFigure 5. **Mixed Logit Model for the Unforced Choice Dataset; Gift Attribute Was Coded as a Continuous Variable **eTable 7. **Mixed Logit Model Results for the Forced Choice Dataset, With Gift Attribute as a Categorical Variable **eTable 8. **Mixed Logit Model Results for the Unforced Choice Dataset, With Gift Attribute as a Categorical Variable **eTable 9. **Relative Importance Derived From Mixed Logit Model Results **eTable 10. **Mixed Logit Model Results for the Unforced Choice Dataset, With Gift Attribute Coded as a Continuous Variable **eTable 11. **Willingness-To-Discard Values for Level Changes in Each Attribute **eTable 12. **Results of Mixed Logit Model With Main Effects and Interactions **eFigure 6. **Attribute Relative Importance Through Subgroup Analysis by Age **eFigure 7. **Attribute Relative Importance Through Subgroup Analysis by Gender **eFigure 8. **Attribute Relative Importance Through Subgroup Analysis by Education **eFigure 9. **Attribute Relative Importance Through Subgroup Analysis by Donation Experience **eFigure 10. **Attribute Relative Importance Through Subgroup Analysis by Volunteer Type
eReferences

Supplement 2.
Data Sharing Statement
