# Error in Bylines

**DOI:** 10.1001/jamanetworkopen.2024.0325

**Published:** 2024-02-07

In the Original Investigation titled “Blood Donors’ Preferences Toward Incentives for Donation in China,”^1^ published June 14, 2023, and in the Invited Commentary titled “Genome Sequencing for Newborn Screening—An Effective Approach for Tackling Rare Diseases,”^2^ published September 1, 2023, there were errors in the bylines. Shan Jiang’s degree should have been listed as an MSc. These articles have been corrected.^1,2^

## References

[1] Wang Y, Zhai P, Jiang S, Li C, Li S. Blood donors’ preferences toward incentives for donation in China. JAMA Netw Open. 2023;6(6):e2318320. doi:10.1001/jamanetworkopen.2023.18320 37314802 PMC10267764

[2] Jiang S, Wang H, Gu Y. Genome sequencing for newborn screening—an effective approach for tackling rare diseases. JAMA Netw Open. 2023;6(9):e2331141. doi:10.1001/jamanetworkopen.2023.31141 37656463

