# Supplementary material for: Blood Donors' Preferences Toward Incentives for Donation in China
Source: JAMA Netw Open. 2023 Jun 14;6(6):e2318320. doi: 10.1001/jamanetworkopen.2023.18320 (PMC10267764; doi:10.1001/jamanetworkopen.2023.18320)
Supplement: Supplement 1. — eTable 1. Literature Search Strategy eTable 2. Characteristics of Participants of Interviews eTable 3. Quotations Describing Blood Donors’ Perspectives on Candidate Attributes From Interviews eTable 4. Attributes and Levels Development From the Focus Group eFigure 1. Example Choice Task in the Original Chinese Version eAppendix. Supplementary Methods eTable 5. Multinomial Logit Model Results in the Pilot Study eTable 6. Multinomial Logit Models Result of Dominant Preference eFigure 2. Mixed Logit Model for the Forced Choice Dataset; Gift Attribute Was Coded as a Categorical Variable eFigure 3. Mixed Logit Model for the Unforced Choice Dataset; Gift Attribute Was Coded as a Categorical Variable eFigure 4. Mixed Logit Model for the Forced Choice Dataset; Gift Attribute Was Coded as a Continuous Variable eFigure 5. Mixed Logit Model for the Unforced Choice Dataset; Gift Attribute Was Coded as a Continuous Variable eTable 7. Mixed Logit Model Results for the Forced Choice Dataset, With Gift Attribute as a Categorical Variable eTable 8. Mixed Logit Model Results for the Unforced Choice Dataset, With Gift Attribute as a Categorical Variable eTable 9. Relative Importance Derived From Mixed Logit Model Results eTable 10. Mixed Logit Model Results for the Unforced Choice Dataset, With Gift Attribute Coded as a Continuous Variable eTable 11. Willingness-To-Discard Values for Level Changes in Each Attribute eTable 12. Results of Mixed Logit Model With Main Effects and Interactions eFigure 6. Attribute Relative Importance Through Subgroup Analysis by Age eFigure 7. Attribute Relative Importance Through Subgroup Analysis by Gender eFigure 8. Attribute Relative Importance Through Subgroup Analysis by Education eFigure 9. Attribute Relative Importance Through Subgroup Analysis by Donation Experience eFigure 10. Attribute Relative Importance Through Subgroup Analysis by Volunteer Type eReferences [file jamanetwopen-e2318320-s001.pdf]

Supplemental Online Content

Wang Y, Zhai P, Jiang S, Li C, Li S. Blood donors' preferences toward incentives for donation in China. *JAMA Netw Open*. 2023;6(6):e2318320. doi:10.1001/jamanetworkopen.2023.18320

eTable 1. Literature Search Strategy
eTable 2. Characteristics of Participants of Interviews
eTable 3. Quotations Describing Blood Donors' Perspectives on Candidate Attributes From Interviews
eTable 4. Attributes and Levels Development From the Focus Group
eFigure 1. Example Choice Task in the Original Chinese Version
eAppendix. Supplementary Methods
eTable 5. Multinomial Logit Model Results in the Pilot Study
eTable 6. Multinomial Logit Models Result of Dominant Preference
eFigure 2. Mixed Logit Model for the Forced Choice Dataset; Gift Attribute Was Coded as a Categorical Variable
eFigure 3. Mixed Logit Model for the Unforced Choice Dataset; Gift Attribute Was Coded as a Categorical Variable
eFigure 4. Mixed Logit Model for the Forced Choice Dataset; Gift Attribute Was Coded as a Continuous Variable
eFigure 5. Mixed Logit Model for the Unforced Choice Dataset; Gift Attribute Was Coded as a Continuous Variable
eTable 7. Mixed Logit Model Results for the Forced Choice Dataset, With Gift Attribute as a Categorical Variable
eTable 8. Mixed Logit Model Results for the Unforced Choice Dataset, With Gift Attribute as a Categorical Variable
eTable 9. Relative Importance Derived From Mixed Logit Model Results
eTable 10. Mixed Logit Model Results for the Unforced Choice Dataset, With Gift Attribute Coded as a Continuous Variable
eTable 11. Willingness-To-Discard Values for Level Changes in Each Attribute
eTable 12. Results of Mixed Logit Model With Main Effects and Interactions
eFigure 6. Attribute Relative Importance Through Subgroup Analysis by Age
eFigure 7. Attribute Relative Importance Through Subgroup Analysis by Gender
eFigure 8. Attribute Relative Importance Through Subgroup Analysis by Education
eFigure 9. Attribute Relative Importance Through Subgroup Analysis by Donation Experience
eFigure 10. Attribute Relative Importance Through Subgroup Analysis by Volunteer Type
eReferences

© 2023 Wang Y et al. *JAMA Network Open.*

This supplemental material has been provided by the authors to give readers additional information about their work.

© 2023 Wang Y et al. *JAMA Network Open.*

Attributes and Levels Development

1. Literature review

Literature search strategy

We searched PubMed and Web of Science for articles in English. The time window is from January 1, 2000, to June 30, 2020.

eTable 1. Literature Search Strategy

Database	Date	Search strategy	Results
PubMed	Jan 2000 — Jun 2020	((((((((incentive[Title/Abstract]) OR (motivation[Title/Abstract])) OR (intention[Title/Abstract])) OR (attitude[Title/Abstract])) OR (barrier[Title/Abstract])) OR (preference[Title/Abstract])) AND (blood donation[Title/Abstract]))) AND ((review[Title/Abstract]) OR (meta-analysis[Title/Abstract]))	27
Web of Science	Jan 2000 — Jun 2020	(TS=incentive OR TS=motivation OR TS=intention OR TS=attitude OR TS=barrier OR TS=preference) AND (TS=blood donation) AND (TS=review OR (TS=meta-analysis)	205

Literature screening

After removing the duplicates, we screened the literature firs by titles and abstracts and then by full-text assessment. In the step of full-text assessment, the exclusion criteria included: (1) the article was not a narrative review or meta-analysis; (2) the

© 2023 Wang Y et al. *JAMA Network Open.*

article was irrelevant with blood donation incentives; and (3) the full text was not available.

2. Interviews

The eTable 2 presented the sociodemographic characteristics of the 24 blood donors participated in the in-depth interview. All interviews were audio-recorded and professionally transcribed verbatim. After analysis of the interview transcripts, we extracted nine candidate attributes, as shown in eTable 3.

eTable 2. Characteristics of Participants of Interviews

	N	%
Sex		
Male	17	70.8
Female	7	29.2
Age, y		
18-24	9	37.5
25-34	6	25.0
35-44	3	12.5
45-55	6	25.0
Education		
Secondary school or below	5	20.9
High school	2	8.3

© 2023 Wang Y et al. *JAMA Network Open.*

Undergraduate diploma	15	62.5
Graduate diploma or above	2	8.3

© 2023 Wang Y et al. *JAMA Network Open.*

eTable 3. Quotations Describing Blood Donors' Perspectives on Candidate Attributes From Interviews

Attribute	Quotation
Health examination	"Well, it's better to have (health check), and because of that, I know more about my health status. "<br>"Because I didn't learn my blood type … (this time) I mainly check my body to see if my blood indicators are normal. I came over to check it out and offered help by the way. "<br>"However, if my parents had the chance, I might be more encouraged. My mom is not well and I hope she can have a general check-up. "
Donation experience	"This is my first blood donation and I was a little nervous at first. However, nurses and other professionals provided prompt guidance and care for some possible discomfort. I feel very good now. "<br>"I think the donation service is great. While donating blood, the nurse felt my hands were cold and gave me a cup of hot water. She is such a thoughtful girl. "

© 2023 Wang Y et al. *JAMA Network Open.*

	“If the donation experience was bad, I might consider whether to donate next time. Fortunately, it was a great experience. I will be back. ”
Blood recipients	“I know that I and my family can use blood for free after donating blood. Other details are unclear. ”<br>“That is, if you donate blood twice (or more), you can use it for free when you really need it in the future. ”<br>"I think, on the one hand, it can provide some degree of protective effects for parents and children in the future. It may be useful, or it may not. ”
Honor for donation	“It's important, because I am proud to have a blood donation certificate, and it makes me happy all day. ”<br>“It does not need to be grand, as long as there is evidence that you have done it. ”<br>“I prefer the donation certificate for COVID-19 pandemic, which makes me feel like I’m supporting the front. ”
Total time	“The total time is very important. Because if the donation center is far away, we (college students) may lack motivation.

© 2023 Wang Y et al. *JAMA Network Open.*

	On campus, we are more active. ”
	“It mostly depends on my time. If it's too far, I probably won't go specifically to donate. ”
	“I have to work. I have no rest time on weekday. Occasionally a day off, I rarely choose to donate blood alone. The mobile blood collection cart is convenient. It is my first choice. ”
Gift	“It’s so cute. I didn't expect a plush toy. I didn't know I would receive a small gift. ”
	“I didn't think about gifts, because I didn't come for them. I think it's okay to receive a gift, or not. ”
	“I will take this gift home for my children. Such gifts (dolls) are not needed at our age. So, we can keep it as a souvenir or give it to our children to nurture their dedication from an early age. ”
Inform usage	“I feel that this message will motivate to give blood. It means a lot to me once I know that I have given something, and even saved a life. ”
	“If I know that my blood is used where it is needed, I will definitely (100%) donate blood next time! ”

© 2023 Wang Y et al. *JAMA Network Open.*

	"I would still like to know about it (the blood usage) if there is a chance, which could potentially give me a lot of encouragement. "
Follow-up	"SMS. I have no time to answer the phone at work. If I see the message, I will reply later. "<br>"I think texting is better. It is a good way to express you concern . If you choose to make a call, the other party may be inconvenient to answer. Better to text. "<br>"I don't care about regular return visits. In fact, I don't need it. If you have to do, we will certainly support your work. But the format (of regular return visits) doesn't matter. "
Three-free policy	"I have never heard of it. What's that (three-free policy)? "<br>"I don't know, but it (three-free policy) might encourage donation. "

© 2023 Wang Y et al. *JAMA Network Open.*

3. Focus Group

Four survey practitioners and four blood donation professionals were invited to participate in the focus group. With the feedback from the focus group, we were able to refine the definition of the attributes and specify their levels (eTable 4).

eTable 4. Attributes and Levels Development From the Focus Group

Attribute	Level	Definition
Health examination	Blood screening<br>Standard examination<br>Comprehensive examination	A free health screening.<br>Routine blood screening for donors.<br>(2) Collection agency provides simple physical examinations for eligible donors (above 1000 ml).<br>(3) Collection agency provides comprehensive hospital examinations for eligible donors (above 2000 ml).
Donation experience	Poor<br>Not bad<br>Good	Comprehensive evaluation of a donation activity.
Blood	Self	Recipients of blood donations.

© 2023 Wang Y et al. *JAMA Network Open.*

recipients	Self plus immediate relatives<br>Self plus immediate/collateral relatives	
Honor for donation	Badge/Certificate<br>Workplace/School recognition<br>Government recognition	Different level of recognition based on the cumulative blood donations.
Total time	120 min<br>90 min<br>60 min	Total time for a donation activity, including travel, queue, donation and observation time.
Gift	RMB 20<br>RMB 40<br>RMB 60	Receipt of small gifts with limited value.

© 2023 Wang Y et al. *JAMA Network Open.*

4. Pilot Testing

After the identification of the attributes and levels, we developed a questionnaire and conducted a pilot study in Jinan, Shandong, in December 2021. The purpose was to verify the comprehensibility and acceptability of the questionnaire. Convenience sampling was used for the pilot study to recruit 86 blood donors. Data were coded as dummy variables and analyzed using STATA version 15.1 (StataCorp LP). Based on the results (eTable 5) and feedback during the pilot study, we made modifications based on feedback to enhance clarity.

© 2023 Wang Y et al. *JAMA Network Open.*

Example Choice Task

eFigure 1. Example Choice Task in the Original Chinese Version

	激励方案 1	激励方案 2
健康检查	全面健康体检	仅献血常规检查
用血优惠	未来其他人优先用血	未来直系亲属优先用血
献血表彰	单位/学校表彰	政府/国家表彰
行程时间	从家/工作地点到最近献血站需 60 分钟行程时间	从家/工作地点到最近献血站需 30 分钟行程时间
小礼物	价值 40 元的小礼物	价值 60 元的小礼物
您更偏好哪个激励方案？	○	○
现实生活中，您是否会接受所选激励方案？	接受 ○	不接受 ○

© 2023 Wang Y et al. *JAMA Network Open.*

Translated version:

Factors	Profile 1	Profile 2
Health Examination	Comprehensive examination	Blood test only
Blood recipients	Future blood recipients unknown	Future blood recipients are immediate relatives
Honor for donation	Honors or recognition from workplace (or school for student donors)	Honors or recognition from the central government
Travel time	60-minute travel time from home/workplace to the nearest blood donation station	30-minute travel time from home/workplace to the nearest blood donation station
Gift	Gift with a monetary value equivalent to RMB 40	Gift with a monetary value equivalent to RMB 60
Which incentive program do you prefer?	◯	◯
If the incentive program you just chose exists in your city, will you accept such an incentive program?	Yes ◯	No ◯

© 2023 Wang Y et al. *JAMA Network Open.*

eAppendix. Supplementary Methods

The DCE was conducted in Shandong between January and April 2022, with the approval from the Ethics Committee of Centre for Health Management and Policy Research, Shandong University (Reference No. ECSHCMSDU20210901). Prior to participating in the survey, all respondents provided their informed consent. Our study adhered to the checklist developed by the International Society for Pharmacoeconomics and Outcomes Research (ISPOR) for good practices pertaining to conjoint analysis in healthcare.[1]

Identification of Attributes and Levels

The DCE commenced with the identification of attributes of the non-monetary incentives for blood donation and defining their respective levels. We primarily extracted the factors from the literature.[2-6] This was followed by in-depth interviews with 24 blood donors and a focus group meeting with four survey practitioners and four blood donation professionals in Jinan, Shandong, from October 2020 to May 2021, to validate the attributes derived from literature and finalize their levels. Ultimately, the DCE questionnaire included five attributes: 1) health examination types; 2) designated blood donation recipients; 3) donation honor; 4) travel time to donation stations; and 5) monetary value of donation gifts. Table 1 provides detailed attribute and level descriptions.

Experimental Design and Questionnaire Development

© 2023 Wang Y et al. *JAMA Network Open.*

A fractional factorial design was executed using SAS version 9.4 (SAS Institute Inc.), resulting in 18 choice tasks. To mitigate cognitive burden for respondents,[7] the 18 tasks were divided into three blocks of six tasks each, with respondents randomized into one of the three blocks. To obtain sufficient information about respondents' preferences and reflect real-world conditions where one could choose not to donate blood any longer, we employed a dual response choice design.[8] Each choice task contained a forced and unforced choice task (eFigure 1). In the forced task, respondents made a choice between two hypothetical profiles, each containing levels respectively from the five attributes. The unforced task provided an opt-out option, requiring respondents to choose between the chosen profile from the forced task and the opt-out option.

The questionnaire comprised an introduction to the research background and attribute definitions, six DCE choice tasks, and sociodemographic inquiries. Before the formal choice tasks, a practice task familiarized respondents with subsequent choice tasks. A duplicated choice task was inserted to ensure respondent attentiveness and consistent choices (i.e., consistency test). Collected sociodemographic information encompassed gender, age, education, occupation, donation experience, donation volume, and volunteer type. Following the questionnaire finalization, we conducted a pilot testing in December 2021 to verify the comprehensibility and acceptability of the questionnaire, making modifications based on feedback to enhance clarity.

Data Collection

© 2023 Wang Y et al. *JAMA Network Open.*

Data were collected from January 1 to April 30, 2022. Eligible participants were blood donors aged between 18 and 60 who had donated within the preceding 12 months. To ensure sample representativeness for the donor population in Shandong, participants were recruited from three cities—Yantai, Jinan, and Heze—located in the eastern, central, and western regions of the province, representing diverse socioeconomic strata. In each city, a blood donation station and a mobile donation vehicle were selected. A convenience sampling approach was employed to recruit donors who visited the selected donation facilities when we surveyed. The sampling process combined predetermined gender and age quotas based on the 2018 China Report on Blood Safety to enhance sample representativeness.[9]

Based on the completion times in the pilot tests, the minimum completion time for the questionnaire was determined to be 120 seconds. If a respondent chose different responses in the two duplicated choice tasks (consistency test), and submitted the questionnaire within 120 seconds, they were considered for removal in the final analysis.

Trained interviewers conducted face-to-face data collection at recruitment locations. The minimum sample size required for ensuring statistical power was determined by the rule of thumb.[10] Considering population size disparities among the three cities, twice the number of donors were recruited in Jinan compared to Yantai and Heze. The interviewers alternated between the three versions of questionnaire during interviews

© 2023 Wang Y et al. *JAMA Network Open.*

to balance the sample size distributed between the three versions.

Sample size

We determined the sample size by Orme's rule of thumb. The rule proposed by Johnson and Orme suggests that the sample size required for the main effects depends on the number of choice tasks (t), the number of alternatives (a), and the number of analysis cells (c) according to the following equation:

$$N > 500c/(t \times a)$$

when considering main effects, 'c' is equal to the largest number of levels for any of the attributes. When considering all two-way interactions, 'c' is equal to the largest product of levels of any two attributes.[10]

Statistical Model

Two mixed logit (MIXL) models were employed to analyze the forced choice data and the unforced choice data, respectively, in STATA version 15.1 (StataCorp LP), estimated via the simulated maximum likelihood approach.[11,12] An alternative-specific constant (ASC) was included in the MIXL for unforced data, indicating the utility generated by the opt-out option relative to the non-opt-out options. To ensure comparability between the models for forced and unforced data, we constrained the ASC in the MIXL of forced data to be 0.[13,14] The categorical attributes were effects-

© 2023 Wang Y et al. *JAMA Network Open.*

coded, with reference levels designated for each attribute. We assumed the parameters of attribute levels followed a normal distribution. Parameter estimations were calculated relative to the reference level within each attribute. The mean of a parameter signified the average preference value (called "part-worth utility") that donors attributed to a specific attribute level, and the standard deviation characterized the heterogeneity of the preference value among donors. To ensure the reliability of the parameter estimates, we iteratively estimated the MIXL model by incrementally increasing the number of random draws by 500, starting from 50 draws. Estimation stability was attained when 2500 draws were used, resulting in our final estimates.

Attribute Relative Importance

The attribute relative importance was calculated using the model estimates of unforced choice dataset through a commonly used rescaling method.[15] The relative importance of each attribute was determined by dividing the range of coefficients within the attribute by the sum of all attribute ranges, subsequently rescaled on a 1-100 range. The highest value denoted the most important attribute perceived by the respondents.

Dominant Preference Examination

Dominant preference refers to respondent's choices being determined by one attribute, i.e., they consistently make choices based solely on the levels of one attribute between the two alternatives in a choice task.[16] Respondents with dominant preference are not making trade-offs between attributes, which hampers the analysis for the relative

© 2023 Wang Y et al. *JAMA Network Open.*

importance between attributes.[16] We applied non-parametric[16] and parametric[17] approaches to test for dominant preference. The former examined whether respondents' choices exhibited a pattern, i.e., the chosen alternatives for the seven tasks were entirely consistent with the alternatives with a higher level of a certain attribute relative to the other alternative in all seven questions. The parametric approach compared the estimated coefficients of attribute levels between a model that included all attributes and a reduced model that included only one attribute. The significant differences between the coefficients indicated the existence of dominant preference caused by that attribute. We iteratively placed each attribute into the reduced model and compared the model estimates with the full model.

Interaction

All potential interactions between the characteristics of respondents and attribute levels were scrutinized through the use of multinomial logit model (MNL).[14] Interaction terms were selected using a backward selection method, based on the contribution of each term to model fit. The log-likelihood ratio test was employed to compare the model specifications and a reduced model with one interaction term removed. If the removed term influenced the model fit significantly, the term was retained. After the identification of interaction terms, we simulated a MIXL model including the interactions to quantify the preference values that respondents placed on the interaction terms.

Willingness-To-Discard

© 2023 Wang Y et al. *JAMA Network Open.*

By coding the attribute “Gift” as a continuous variable in the model, the marginal rate of substitution between gift value and other aspects of non-monetary incentives could be calculated, interpreted as donor’s willingness-to-discard (WTD) gifts for the improvement of other incentive factors. By comparing the WTD values for the improvement of four factors—health examination, blood recipient, honor recognition, and travel time—policy implications were generated for efficiently augmenting non-monetary incentives to elevate the utility of donors.

Scenario Analysis

The prevailing incentive profile in Shandong comprised providing blood test and certificates to donors, designating donors themselves as the recipients of their donation, and an average 90-minute travel time to the nearest donation station. We constructed hypothetical incentive profiles where the attributes were improved compared with the base profile. The comparisons between the hypothetical profiles and the base profile constituted the scenario analysis. The outcome of scenario analysis was denoted by the predicted uptake for each hypothetical profile relative to the base case, defined as the percentage of donors amenable to supporting the new incentive profile, which showcased how the improvement endeavour could motivate donors. The 95% confidence intervals for predicted uptake were generated using delta method.[18]

Subgroup analysis

© 2023 Wang Y et al. *JAMA Network Open.*

Employing individual-level preferences, we conducted subgroup analyses by comparing the attribute relative importance between different groups of respondents. The variables we used to categorize respondents included age, gender, education level, first-time donor, and individual volunteer.

© 2023 Wang Y et al. *JAMA Network Open.*

Model specifications

1. Multinomial Logit model

Multinomial logit models are used to model relationships between a polytomous response variable and a set of regressor variables.[19] Multinomial logit was shown by McFadden to be consistent with random utility theory. Using random utility theory, the utility associated with an alternative or profile is assumed to be a function of observed characteristics (attribute levels) and unobserved characteristics of the alternative. The utility function is specified as an indirect utility function defined by the attribute levels in the alternative plus a random error term reflecting the researcher's inability to perfectly measure utility:

$$\mathrm{U}i = V(\beta, Xi) + \varepsilon i$$

where V is a function defined by the attribute levels for alternative i, εi is a random error term, Xi is a vector of attribute levels defining alternative i, and β is a vector of estimated coefficients. Each estimated coefficient is a preference weight and represents the relative contribution of the attribute level to the utility that respondents assign to an alternative. In Multinomial logit, εi is assumed to follow an independently and identically distributed type 1 extreme-value distribution.

The assumption of the extreme-value distribution of εi results in a logit model:

$$\Pr(choice = i) = \frac{e^{V(\beta, Xi)}}{\sum_j e^{V(\beta, Xj)}}$$

© 2023 Wang Y et al. *JAMA Network Open.*

where *V(β, Xi)* is the observed portion of the function for alternative *i*, and *i* is one alternative among a set of *j* alternatives. Simply stated, the probability of choosing alternative *i* is a function of both the attribute levels of alternative *i* and the attribute levels of all other profiles presented in a choice task.[20]

2. Mixed logit model

Like conditional logit, mixed logit, MXL (also called "random parameters logit, RPL") is a method that assumes that the probability of choosing a profile from a set of alternatives is a function of the attribute levels that characterize the alternatives and a random error term that adjusts for individual-specific variations in preferences. Unlike conditional logit that estimates only a set of coefficients capturing the mean preference weights of the attribute levels, MXL yields both a mean effect and a standard deviation of effects across the sample. That is, MXL explicitly assumes that there is a distribution of preference weights across the sample reflecting differences in preferences among respondents, and it models the parameters of that distribution for each attribute level. The choice probability of the MXL model is as follows:

$$\Pr(choice_n = i) = \frac{e^{V(\tilde{\beta}_n, X_i)}}{\sum_j e^{V(\tilde{\beta}_n, Xj)}}$$

where *n* indexes respondents in the sample, $\tilde{\beta}_{\mathrm{n}} = f(\beta, \sigma \mid V_n)$, β and σ are parameters to be estimated on the basis of systematic variations in preferences across individuals in the sample given the variable V_n characterizing individual-specific

© 2023 Wang Y et al. *JAMA Network Open.*

heterogeneity, and *f( )* is a function determining the distribution of $\tilde{\beta}_n$ across respondents, given parameters *β* and *σ*. Commonly, $\tilde{\beta}$ is assumed to be normally distributed with mean *β* and standard deviation *σ*, which means the following:[20]

$$\begin{aligned}\tilde{\beta}_n &= f(\beta, \sigma \mid V_n) \\ &= \beta + \sigma V_n,\ for V_n \sim N(0,1) \text{across respondents in the sample}\end{aligned}$$

© 2023 Wang Y et al. *JAMA Network Open.*

eTable 5. Multinomial Logit Model Results in the Pilot Study

	β	SE	P value	95% CI	
Constant	-1.35	0.66	0.04	-2.63	-0.06
Gift_con	0.00	0.00	0.92	-0.01	0.01
Honor for donation					
Certificate	0.00	—	—	—	—
Workplace	-0.35	0.17	0.04	-0.67	-0.02
Government	0.34	0.24	0.16	-0.13	0.80
Travel time					
90 min	0.00	—	—	—	—

© 2023 Wang Y et al. *JAMA Network Open.*

60 min	0.43	0.19	0.03	0.05	0.80
30 min	0.84	0.21	0.00	0.42	1.26
Health examination					
Blood	0.00	—	—	—	—
Standard	0.22	0.18	0.23	-0.14	0.59
Comprehensive	0.64	0.22	0.00	0.22	1.07
Blood recipients					
Unknown	0.00	—	—	—	—
Immediate relative	1.19	0.21	0.00	0.79	1.60
Donor	-0.16	0.24	0.51	-0.63	0.31

Note: 1.SE: standard error. 95% CI: confidence interval. Gift_co: Gift_continuous. 2.A total of 86 questionnaires were collected in the pilot testing,

© 2023 Wang Y et al. *JAMA Network Open.*

and 58 respondents who passed the consistency test were included in this table.

© 2023 Wang Y et al. *JAMA Network Open.*

Supplementary results of statistical analysis

eTable 6. Multinomial Logit Models Result of Dominant Preference

Attribute and level	full model	only examination	only recipient	only honor	only travel time	only gift
	Part-worth utility, mean	Part-worth utility, mean	Part-worth utility, mean	Part-worth utility, mean	Part-worth utility, mean	Part-worth utility, mean
Constant	-2.04	-2.07	-2.06	-2.08	-2.07	-2.07
Health Examination						
blood (ref)	-0.13	-0.06				
standard	-0.08	-0.12				
comprehensive	0.22	0.18				

© 2023 Wang Y et al. *JAMA Network Open.*

Blood recipients				
unknown (ref)	-0.45	-0.46		
self	-0.14	-0.13		
family	0.59	0.58		
Honor for donation				
certificate (ref)	0.01		0.04	
workplace	-0.13		-0.13	
government	0.13		0.08	
Travel time				
90 min (ref)	-0.15			-0.11
60 min	0.02			0.01

© 2023 Wang Y et al. *JAMA Network Open.*

30 min	0.13				0.10	
Gift						
RMB 20 (ref)	-0.19					-0.20
RMB 40	-0.01					0.05
RMB 60	0.19					0.15
Log-likelihood	-2720.321	-2961.812	-2788.334	-2972.582	-2973.062	-2956.094
AIC	5462.642	5929.623	5582.668	5951.165	5952.124	5918.189
BIC	5542.184	5951.316	5604.361	5972.858	5973.817	5939.882
n	568	568	568	568	568	568

© 2023 Wang Y et al. *JAMA Network Open.*

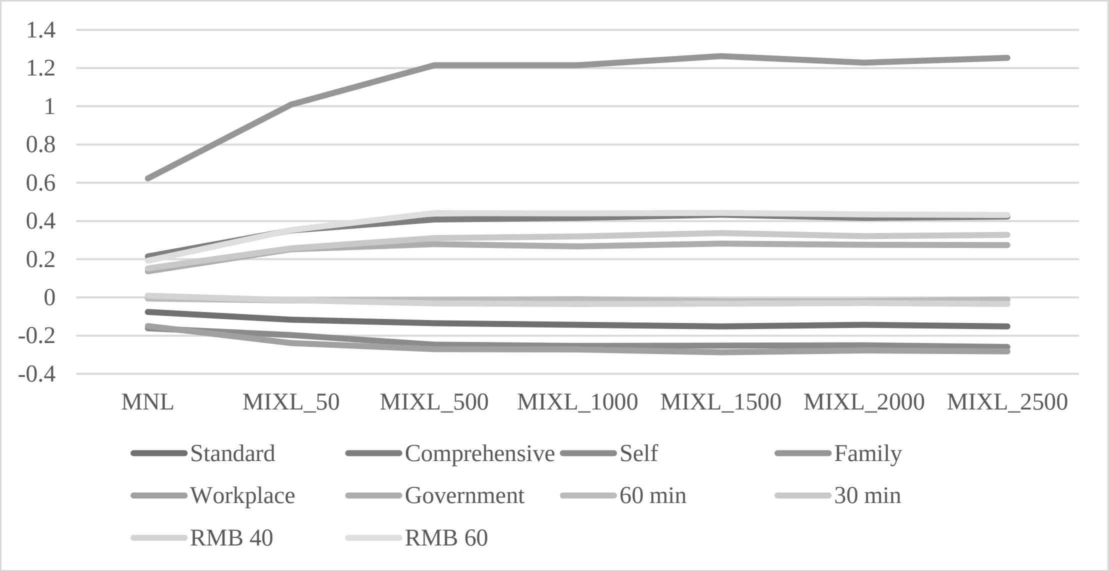

eFigure 2. Mixed Logit Model for the Forced Choice Dataset; Gift Attribute Was Coded as a Categorical Variable

Note: MNL, multinomial logit model; MIXL, mixed logit model.

© 2023 Wang Y et al. *JAMA Network Open.*

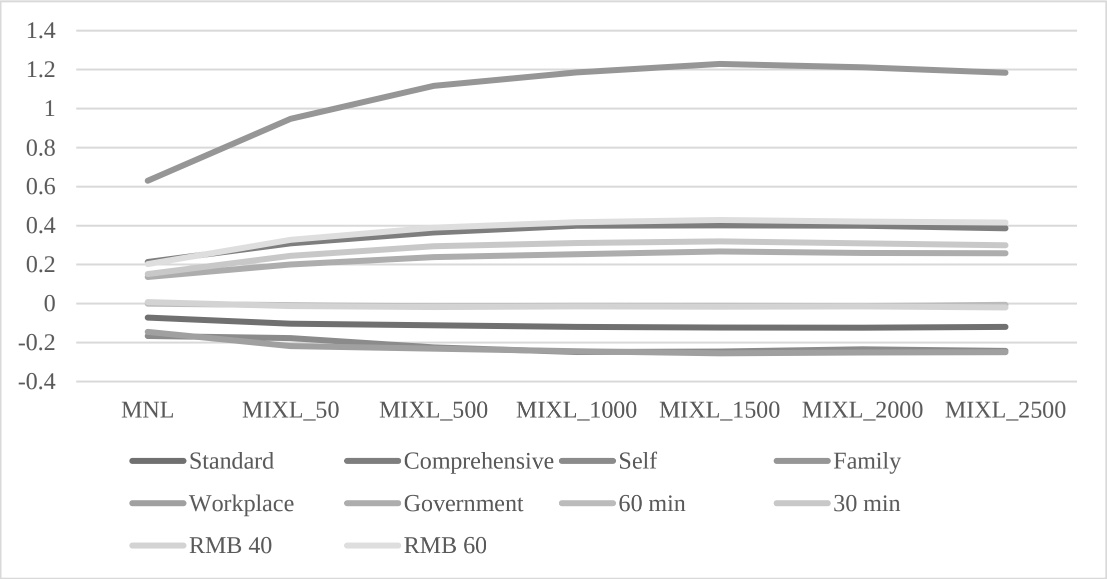

eFigure 3. Mixed Logit Model for the Unforced Choice Dataset; Gift Attribute Was Coded as a Categorical Variable

Note: MNL, multinomial logit model; MIXL, mixed logit model.

© 2023 Wang Y et al. *JAMA Network Open.*

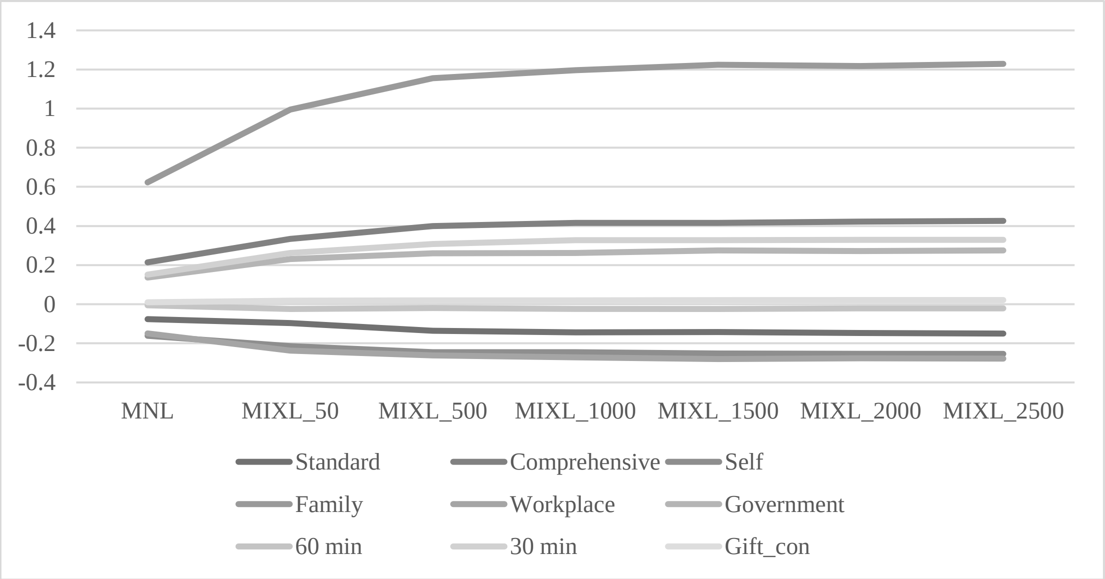

eFigure 4. Mixed Logit Model for the Forced Choice Dataset; Gift Attribute Was Coded as a Continuous Variable

Note: MNL, multinomial logit model; MIXL, mixed logit model.

© 2023 Wang Y et al. *JAMA Network Open.*

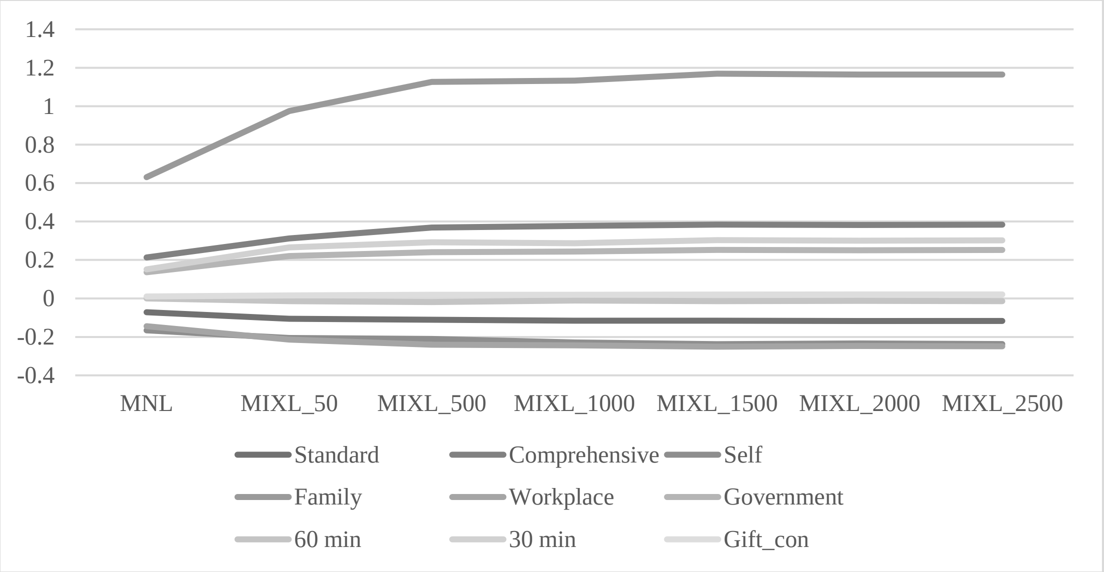

eFigure 5. Mixed Logit Model for the Unforced Choice Dataset; Gift Attribute Was Coded as a Continuous Variable

Note: MNL, multinomial logit model; MIXL, mixed logit model.

© 2023 Wang Y et al. *JAMA Network Open.*

eTable 7. Mixed Logit Model Results for the Forced Choice Dataset, With Gift Attribute as a Categorical Variable

	β	SE	P value	95% CI		SD	P value	Part-worth utility<0
Health examination								
Blood	-0.27	0.07	0.00	-0.41	-0.13	0.44	0.05	1.00
Standard	-0.15	0.06	0.02	-0.28	-0.03	0.03	0.86	0.99
Comprehensive	0.42	0.08	0.00	0.27	0.58	0.42	0.01	0.00
Blood recipients								
Unknown	-0.99	0.14	0.00	-1.27	-0.71	0.10	0.43	1.00
Self	-0.26	0.08	0.00	-0.42	-0.10	1.15	0.00	1.00
Family	1.25	0.16	0.00	0.95	1.56	1.04	0.00	0.00
Honor for donation								

© 2023 Wang Y et al. *JAMA Network Open.*

Certificate	0.01	0.07	0.90	-0.12	0.14	1.05	0.00	0.45
Workplace	-0.28	0.07	0.00	-0.41	-0.15	0.53	0.00	1.00
Government	0.27	0.07	0.00	0.14	0.41	0.52	0.00	0.00
Travel time								
90 min	-0.32	0.07	0.00	-0.46	-0.17	0.67	0.02	1.00
60 min	-0.01	0.06	0.85	-0.13	0.11	0.05	0.86	0.57
30 min	0.33	0.08	0.00	0.18	0.48	0.72	0.00	0.00
Gift								
RMB 20	-0.40	0.08	0.00	-0.56	-0.23	0.69	0.05	1.00
RMB 40	-0.03	0.06	0.56	-0.15	0.08	0.16	0.59	0.72
RMB 60	0.43	0.08	0.00	0.27	0.59	0.53	0.00	0.00

© 2023 Wang Y et al. *JAMA Network Open.*

Note: SE: standard error. SD: standard deviation. 95% CI: confidence interval.

© 2023 Wang Y et al. *JAMA Network Open.*

eTable 8. Mixed Logit Model Results for the Unforced Choice Dataset, With Gift Attribute as a Categorical Variable

	β	SE	P value	95% CI		SD	P value	Part-worth utility<0
Constant	-9.82	1.42	0.00	-12.60	-7.04	6.10	0.00	—
Health examination								
Blood	-0.27	0.07	0.00	-0.40	-0.14	0.31	0.15	1.00
Standard	-0.12	0.06	0.04	-0.23	0.00	0.00	0.98	0.98
Comprehensive	0.39	0.07	0.00	0.25	0.52	0.32	0.06	0.00
Blood recipients								
Unknown	-0.94	0.12	0.00	-1.17	-0.71	0.14	0.25	1.00
Self	-0.24	0.08	0.00	-0.39	-0.10	1.09	0.00	1.00
Family	1.18	0.12	0.00	0.95	1.42	0.94	0.00	0.00

© 2023 Wang Y et al. *JAMA Network Open.*

Honor for donation								
Certificate	-0.01	0.06	0.90	-0.13	0.11	0.93	0.00	0.55
Workplace	-0.25	0.06	0.00	-0.37	-0.13	0.50	0.00	1.00
Government	0.26	0.06	0.00	0.13	0.38	0.43	0.00	0.00
Travel time								
90 min	-0.29	0.07	0.00	-0.42	-0.16	0.61	0.03	1.00
60 min	-0.01	0.06	0.91	-0.12	0.11	0.02	0.93	0.55
30 min	0.30	0.07	0.00	0.17	0.43	0.64	0.00	0.00
Gift								
RMB 20	-0.40	0.08	0.00	-0.55	-0.24	0.75	0.00	1.00

© 2023 Wang Y et al. *JAMA Network Open*.

RMB 40	-0.02	0.06	0.73	-0.13	0.09	0.23	0.24	0.64
RMB 60	0.42	0.07	0.00	0.27	0.56	0.51	0.00	0.00

Note: SE: standard error. SD: standard deviation. 95% CI: confidence interval.

© 2023 Wang Y et al. *JAMA Network Open.*

eTable 9. Relative Importance Derived From Mixed Logit Model Results

	Forced choice				Unforced choice			
	β	SE	P value	95% CI	β	SE	P value	95% CI
Health examination	0.14	0.02	0.00	0.10, 0.18	0.14	0.02	0.00	0.10, 0.18
Blood recipients	0.45	0.03	0.00	0.40, 0.51	0.45	0.03	0.00	0.40, 0.51
Honor for donation	0.11	0.02	0.00	0.08, 0.15	0.11	0.02	0.00	0.07, 0.15
Travel time	0.13	0.02	0.00	0.09, 0.17	0.13	0.02	0.00	0.08, 0.17
Gift	0.17	0.02	0.00	0.12, 0.21	0.17	0.02	0.00	0.13, 0.22

Note: SE: standard error. SD: standard deviation. 95% CI: confidence interval.

© 2023 Wang Y et al. *JAMA Network Open.*

eTable 10. Mixed Logit Model Results for the Unforced Choice Dataset, With Gift Attribute Coded as a Continuous Variable

	β	SE	P value	95% CI		SD	P value	Part-worth utility<0
Constant	-8.77	1.34	0.00	-11.39	-6.14	5.88	0.09	—
Health examination								
Blood	-0.27	0.06	0.00	-0.39	-0.14	0.30	0.16	1.00
Standard	-0.12	0.06	0.04	-0.23	0.00	0.00	0.97	0.98
Comprehensive	0.38	0.07	0.00	0.25	0.51	0.30	0.08	0.00
Blood recipients								
Unknown	-0.93	0.11	0.00	-1.14	-0.71	0.16	0.18	1.00
Self	-0.24	0.07	0.00	-0.38	-0.09	1.08	0.00	1.00
Family	1.16	0.11	0.00	0.95	1.38	0.91	0.00	0.00

© 2023 Wang Y et al. *JAMA Network Open.*

Honor for donation								
Certificate	0.00	0.06	0.97	-0.12	0.12	0.04	0.74	0.52
Workplace	-0.25	0.06	0.00	-0.36	-0.14	0.49	0.00	1.00
Government	0.25	0.06	0.00	0.13	0.37	0.44	0.00	0.00
Travel time								
90 min	-0.29	0.06	0.00	-0.42	-0.16	0.59	0.01	1.00
60 min	-0.01	0.06	0.80	-0.12	0.10	0.04	0.84	0.60
30 min	0.30	0.07	0.00	0.17	0.44	0.63	0.00	0.00
Gift_con	0.02	0.00	0.00	0.01	0.03	0.02	0.00	0.00

Note: SE: standard error. SD: standard deviation. 95% CI: confidence interval. Gift_con: Gift attribute coded as a continuous variable.

© 2023 Wang Y et al. *JAMA Network Open.*

eTable 11. Willingness-To-Discard Values for Level Changes in Each Attribute

Base profile	Best profile	WTD	P value	95% CI, lower	95% CI, upper
Blood	Comprehensive	32.03	0.00	18.22	45.84
Self	Family	69.18	0.00	46.58	91.78
Certificate	Government	12.54	0.02	2.05	23.03
90 min	30 min	29.20	0.00	16.25	42.15

Note: WTD: willingness-to-discard. 95% CI: confidence interval.

© 2023 Wang Y et al. *JAMA Network Open.*

eTable 12. Results of Mixed Logit Model With Main Effects and Interactions

	β	SE	P value	95% CI		SD	P value
Constant(opt)	-7.98	1.29	0.00	-10.51		5.65	0.00
Health examination							
Blood	-0.58	0.17	0.00	-0.91	-0.25	0.35	0.53
Standard	0.07	0.09	0.47	-0.11	0.25	0.04	0.93
Comprehensive	0.51	0.10	0.00	0.32	0.71	0.39	0.18
Blood recipients							
Unknown	-2.11	0.26	0.00	-2.62	-1.61	2.33	0.00
Self	0.45	0.17	0.01	0.11	0.79	1.27	0.00
Family	1.67	0.15	0.00	1.38	1.95	1.07	0.00

© 2023 Wang Y et al. *JAMA Network Open.*

Honor for donation							
Certificate	0.16	0.15	0.27	-0.13	0.45	0.67	0.10
Workplace	-0.28	0.08	0.00	-0.45	-0.12	0.40	0.06
Government	0.12	0.09	0.17	-0.05	0.30	0.27	0.40
Travel time							
90 min	-0.56	0.16	0.00	-0.88	-0.24	0.89	0.06
60 min	0.22	0.08	0.01	0.06	0.38	0.12	0.79
30 min	0.34	0.12	0.00	0.11	0.57	0.77	0.00
Gift_con	0.02	0.00	0.00	0.01	0.02	0.03	0.00
Male_self_int	-0.48	0.20	0.02	-0.87	-0.08	0.21	0.72

© 2023 Wang Y et al. *JAMA Network Open.*

High school or less_self_int	0.69	0.20	0.00	0.30	1.09	0.73	0.12
First-time donor_30 min_int	0.42	0.17	0.01	0.09	0.76	0.00	1.00

Note: 1.SE: standard error. SD: standard deviation. 95% CI: confidence interval. 2. Gift_co: gift_continuous. Male_self_int: male_self_interaction.

High school_self_int: high school or less_self_interaction. First_30 min_int: first-time donor_30 min_interaction.

© 2023 Wang Y et al. *JAMA Network Open.*

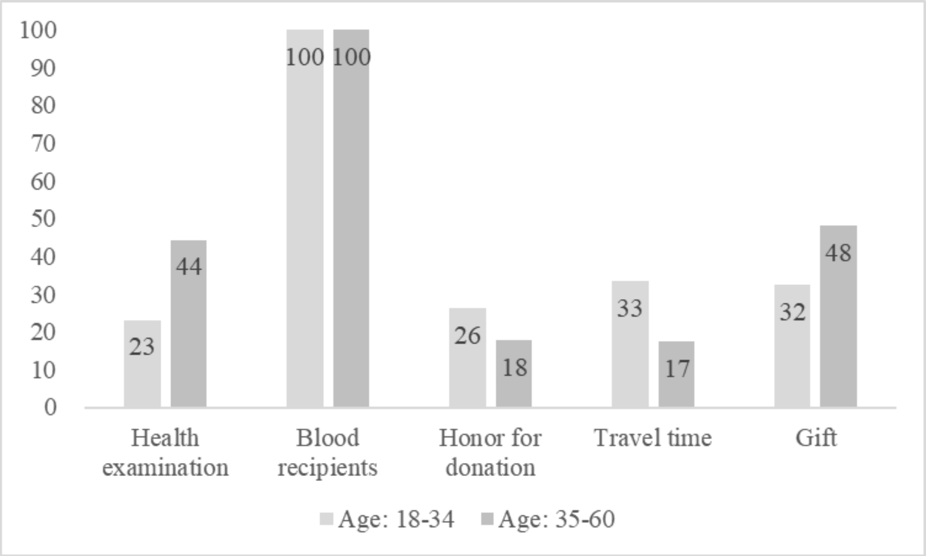

eFigure 6. Attribute Relative Importance Through Subgroup Analysis by Age

© 2023 Wang Y et al. *JAMA Network Open.*

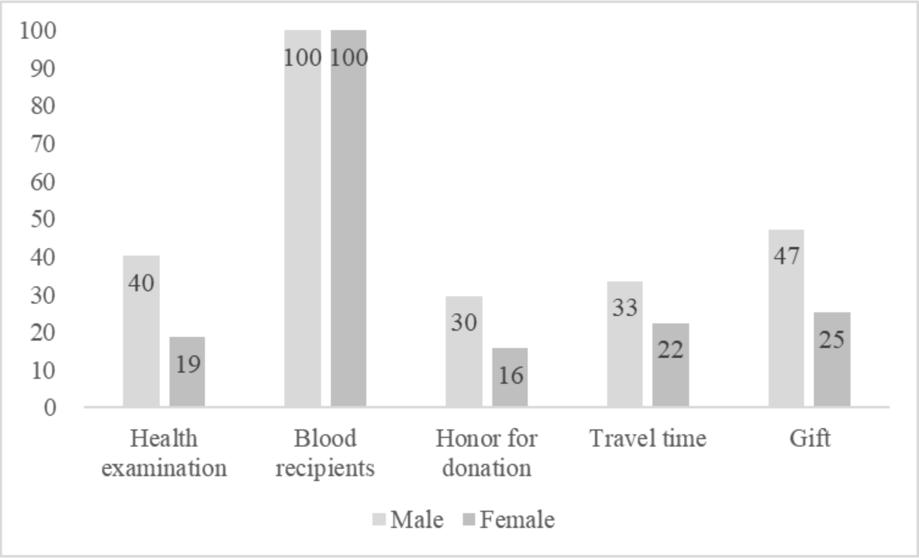

eFigure 7. Attribute Relative Importance Through Subgroup Analysis by Gender

© 2023 Wang Y et al. *JAMA Network Open.*

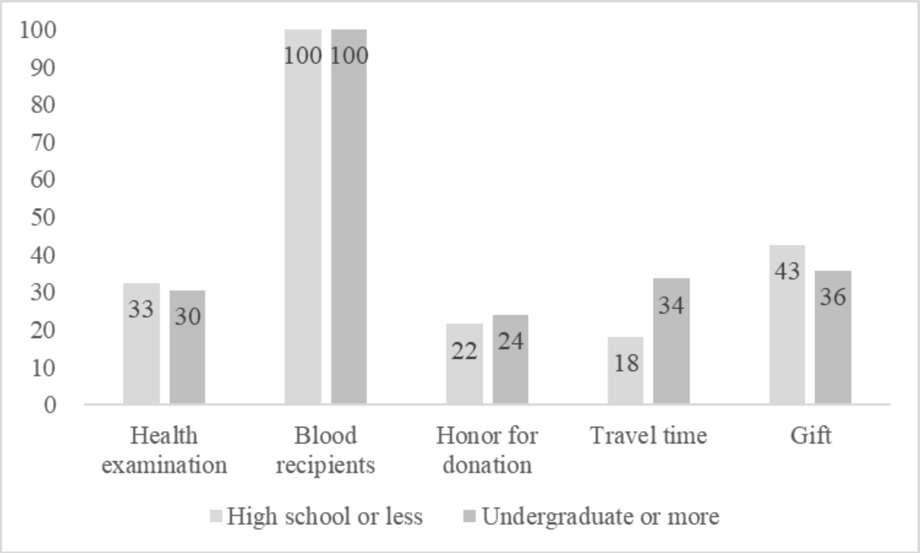

eFigure 8. Attribute Relative Importance Through Subgroup Analysis by Education

© 2023 Wang Y et al. *JAMA Network Open.*

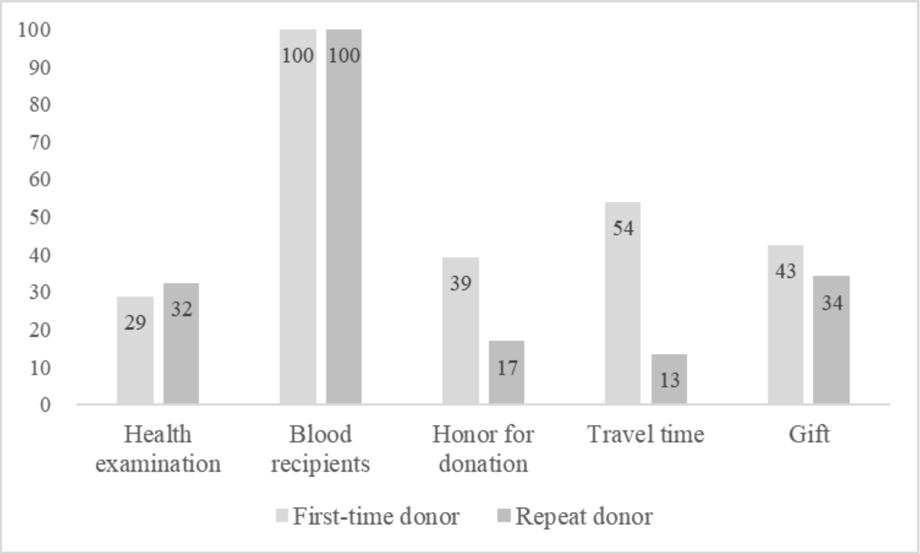

eFigure 9. Attribute Relative Importance Through Subgroup Analysis by Donation Experience

© 2023 Wang Y et al. *JAMA Network Open.*

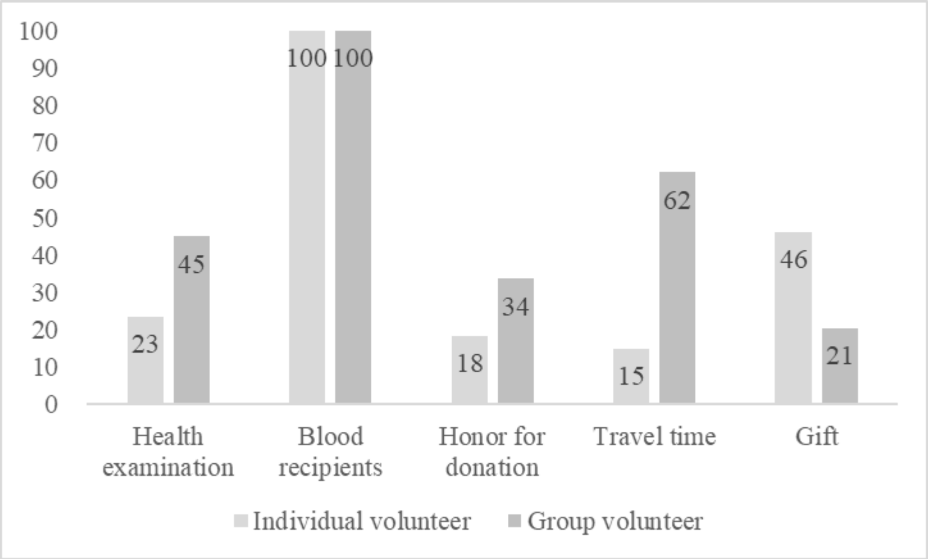

eFigure 10. Attribute Relative Importance Through Subgroup Analysis by Volunteer Type

© 2023 Wang Y et al. *JAMA Network Open.*

eReferences

1. Bridges JF, Hauber AB, Marshall D, et al. Conjoint analysis applications in health--a checklist: a report of the ISPOR Good Research Practices for Conjoint Analysis Task Force. *Value Health*. Jun 2011;14(4):403-13. doi:10.1016/j.jval.2010.11.013

2. Chell K, Davison TE, Masser B, Jensen K. A systematic review of incentives in blood donation. *Transfusion*. Jan 2018;58(1):242-254. doi:10.1111/trf.14387

3. Chmielewski D, Bove LL, Lei J, Neville B, Nagpal A. A new perspective on the incentive-blood donation relationship: partnership, congruency, and affirmation of competence. *Transfusion*. Sep 2012;52(9):1889-900. doi:10.1111/j.1537-2995.2011.03545.x

4. Glynn SA, Williams AE, Nass CC, et al. Attitudes toward blood donation incentives in the United States: implications for donor recruitment. *Transfusion*. Jan 2003;43(1):7-16. doi:10.1046/j.1537-2995.2003.00252.x

5. Sanchez AM, Ameti DI, Schreiber GB, et al. The potential impact of incentives on future blood donation behavior. *Transfusion*. Feb 2001;41(2):172-8. doi:10.1046/j.1537-2995.2001.41020172.x

6. Kasraian L, Maghsudlu M. Blood donors' attitudes towards incentives: influence on motivation to donate. *Blood Transfus*. Apr 2012;10(2):186-90. doi:10.2450/2011.0039-11

7. Jiang S, Gu Y, Yang F, et al. Tertiary hospitals or community clinics? An enquiry into the factors affecting patients' choice for healthcare facilities in urban China. *China Econ Rev*. 2020:101538. doi:10.1016/j.chieco.2020.101538

8. Brazell JD, Diener CG, Karniouchina E, Moore WL, Séverin V, Uldry P-F. The no-choice option and dual response choice designs. *Market Lett*. 2006;17(4):255-268. doi:10.1007/s11002-006-7943-8

9. National Health Commission of the People's Republic of China. *China's Report on Blood Safety*

© 2023 Wang Y et al. *JAMA Network Open.*

2018. People's Medical Publishing House; 2020.

10. de Bekker-Grob EW, Donkers B, Jonker MF, Stolk EA. Sample Size Requirements for Discrete-Choice Experiments in Healthcare: a Practical Guide. *Patient*. Oct 2015;8(5):373-84. doi:10.1007/s40271-015-0118-z

11. Train KE. *Discrete choice methods with simulation*. Cambridge university press; 2009.

12. Hole AR. Fitting Mixed Logit Models by Using Maximum Simulated Likelihood. *Stata J*. 2007;7(3):388-401. doi:10.1177/1536867x0700700306

13. Mohammadi T, Zhang W, Sou J, Langlois S, Munro S, Anis AH. A Hierarchical Bayes Approach to Modeling Heterogeneity in Discrete Choice Experiments: An Application to Public Preferences for Prenatal Screening. *Patient*. 2019:1-13. doi:10.1007/s40271-019-00402-w PMID - 31814082

14. Jiang S, Anis AH, Cromwell I, et al. Health-care practitioners' preferences for the return of secondary findings from next-generation sequencing: a discrete choice experiment. *Genet Med*. 2020:1-9. doi:10.1038/s41436-020-0927-x PMID - 32820245

15. Krucien N, Watson V, Ryan M. Is Best–Worst Scaling Suitable for Health State Valuation? A Comparison with Discrete Choice Experiments. *Health Econ*. 2017;26(12):e1-e16. doi:10.1002/hec.3459 PMID - 27917560

16. Vaughan L, Bardsley M, Bell D, et al. Health Services and Delivery Research. *Models of generalist and specialist care in smaller hospitals in England: a mixed-methods study*. NIHR Journals Library; 2021.

17. Mohammadi T, Zhang W, Sou J, Langlois S, Munro S, Anis AH. A Hierarchical Bayes Approach to Modeling Heterogeneity in Discrete Choice Experiments: An Application to Public Preferences for Prenatal Screening. *Patient*. Apr 2020;13(2):211-223. doi:10.1007/s40271-019-00402-w

© 2023 Wang Y et al. *JAMA Network Open.*

18. Regier DA, Peacock SJ, Pataky R, et al. Societal preferences for the return of incidental findings from clinical genomic sequencing: a discrete-choice experiment. *Canadian Medical Association Journal*. 2015;187(6):E190-E197.

19. So Y, F. KW. Multinomial logit models. presented at: SUGI 20 conference proceedings; 1995;

20. Hauber AB, González JM, Groothuis-Oudshoorn CG, et al. Statistical Methods for the Analysis of Discrete Choice Experiments: A Report of the ISPOR Conjoint Analysis Good Research Practices Task Force. *Value Health*. Jun 2016;19(4):300-15. doi:10.1016/j.jval.2016.04.004

© 2023 Wang Y et al. *JAMA Network Open.*
