# Supplementary material for: Blood Donors' Preferences Toward Incentives for Donation in China
Source: JAMA Netw Open. 2023 Jun 14;6(6):e2318320. doi: 10.1001/jamanetworkopen.2023.18320 (PMC10267764; doi:10.1001/jamanetworkopen.2023.18320)
Supplement: Supplement 2. — Data Sharing Statement [file jamanetwopen-e2318320-s002.pdf]

Data Sharing Statement

Wang. Blood Donors' Preferences Toward Incentives for Donation in China. *JAMA Netw Open*. Published June 14, 2023. doi:10.1001/jamanetworkopen.2023.18320

Data
Data available: No
